# Restoration of Radial Bowing After Elastic Stable Intramedullary Nailing in Pediatric Forearm Fractures: The Importance of Bowing Location in Addition to Magnitude

**DOI:** 10.3390/jcm15176743

**Published:** 2026-08-30

**Authors:** Osman Görkem Muratoğlu, Hasan Ceylan, Gökhan Güzel, Fatih Günaydın, Fevzi Mehmet Kuyumcu, Mehmet Ali Talmaç, Cem Yıldırım

**Affiliations:** 1 Department of Orthopaedics and Traumatology, Atlas University Faculty of Medicine, Atlas University Hospital, Istanbul 34203, Türkiye; hasan.ceylan@atlas.edu.tr (H.C.); gokhan.guzel@atlas.edu.tr (G.G.); cem.yildirim@atlas.edu.tr (C.Y.); 2 Department of Orthopaedics and Traumatology, Mersin City Hospital, Mersin 33240, Türkiye; drfatihgunaydin@gmail.com; 3 Department of Orthopaedics and Traumatology, Cam and Sakura City Hospital, Istanbul 34480, Türkiye; fevzimkuyumcu@gmail.com (F.M.K.); drtalmac2@gmail.com (M.A.T.)

**Keywords:** elastic stable intramedullary nailing, pediatric forearm fractures, radial bowing, diaphyseal fractures, radiographic assessment

## Abstract

**Background/Objectives:** Elastic stable intramedullary nailing (ESIN) is widely used for the treatment of pediatric diaphyseal forearm fractures. Although fracture union is commonly achieved, restoration of physiological radial bowing is essential for optimal forearm biomechanics. Most previous studies have focused on bowing magnitude, whereas the anatomical location of maximal radial bowing has received limited attention. **Methods:** This retrospective observational study included pediatric patients aged 4–16 years who underwent ESIN for diaphyseal radius and ulna fractures and had a minimum follow-up of 1 year. Postoperative anteroposterior radiographs were analyzed to determine maximal radial bowing (MRB) and the location of maximal radial bowing (LMRB) using standardized radiographic methods. Simultaneous concordance with the literature-derived MRB (6–9%) and LMRB (55–75%) reference ranges was evaluated as an exploratory composite radiographic outcome. Clinical, radiological, and surgical variables were analyzed, and multivariable logistic regression was performed to identify factors associated with the exploratory composite radiographic outcome. **Results:** A total of 61 patients were included. The mean age was 10.8 ± 2.6 years, and 86.9% were male. Mean MRB was 5.9 ± 0.9%. MRB and LMRB were within the predefined literature-derived reference ranges in 55.7% and 85.2% of patients, respectively, and simultaneous concordance with both ranges was observed in 50.8%. A significant association was identified between MRB and LMRB, indicating that increased bowing magnitude was associated with a more proximally located maximal bow. The injury-to-surgery interval was not independently associated with the exploratory composite radiographic outcome. Despite radiological variability, 95.1% of patients demonstrated excellent or good functional outcomes. **Conclusions:** In this retrospective cohort, combined evaluation of MRB and LMRB provided a descriptive assessment of postoperative radial alignment. Concordance with both predefined reference ranges was associated with less supination loss; however, no individual radiographic parameter showed a significant correlation with functional outcomes.

## 1. Introduction

Pediatric forearm fractures are among the most common long-bone injuries in children, with the majority involving diaphyseal fractures of the radius and ulna. Current concepts emphasize elastic stable intramedullary nailing (ESIN) as a preferred surgical option for unstable fractures because of its minimally invasive nature, preservation of fracture biology, and reliable union rates, while allowing for early functional recovery [1,2]. Nevertheless, successful treatment extends beyond fracture healing alone and requires restoration of native forearm anatomy to achieve optimal biomechanical and functional outcomes.

The radius has a characteristic physiological curvature, referred to as radial bowing, which plays a critical role in forearm biomechanics and rotational movements. Both the magnitude of radial bowing and the anatomical location of its maximum are key determinants of pronation and supination [3]. Inadequate restoration of radial bowing after surgical fixation may alter radius–ulna kinematics and result in residual functional limitations, even when radiographic union is achieved.

Radiographic assessment of radial bowing has been standardized using the modified Schemitsch and Richards technique, which allows for objective evaluation of both bowing magnitude and its location along the radial shaft [4]. Reference values for maximal radial bowing and its anatomical position have been reported in pediatric populations [3,5]. However, despite the widespread use of ESIN, the factors influencing postoperative restoration of radial bowing in pediatric diaphyseal forearm fractures remain incompletely understood. In particular, the relative contribution of patient-related characteristics and surgery-related parameters, such as implant selection and timing of surgical intervention, has not been clearly established in the literature [1,2].

Therefore, the present study aimed to evaluate postoperative restoration of radial bowing in pediatric patients treated with elastic stable intramedullary nailing for diaphyseal forearm fractures by assessing both the amount and the anatomical location of maximal radial bowing. We hypothesized that postoperative radial alignment may be more comprehensively described by a combined evaluation of bowing magnitude and location than by bowing magnitude alone, and that deviations from the predefined radial bow reference ranges may be associated with patient-, fracture-, and surgery-related factors.

## 2. Methods

### 2.1. Study Design and Patients

This retrospective, single-center observational study was conducted in accordance with the Declaration of Helsinki and approved by the local institutional ethics committee. Pediatric patients aged between 4 and 16 years who underwent surgical treatment with elastic stable intramedullary nailing (ESIN) for diaphyseal fractures of both the radius and ulna were retrospectively reviewed. Inclusion criteria were: (1) diaphyseal fractures of the radius and ulna, (2) treatment with ESIN, (3) a minimum follow-up duration of 12 months, and (4) availability of complete anteroposterior (AP) and lateral radiographs obtained postoperatively and at final follow-up. Patients with pathological fractures, Salter–Harris physeal injuries, or insufficient radiological data were excluded from the study. Consecutive pediatric patients who underwent elastic stable intramedullary nailing for diaphyseal forearm fractures between January 2021 and January 2025 were retrospectively identified. Clinical and radiographic data were collected and analyzed in January 2026.

### 2.2. Surgical Technique

All procedures were performed by or under the direct supervision of orthopaedic trauma surgeons with at least five years of experience in trauma surgery. Under general anesthesia, elastic stable intramedullary nailing (ESIN) was performed using a standardized surgical technique. Flexible titanium nails were inserted through metaphyseal entry points under fluoroscopic guidance. Nail diameters were selected according to the intramedullary canal diameter to achieve balanced elastic fixation and optimal three-point support. Closed reduction was attempted in all cases. Limited open reduction was performed only when satisfactory alignment could not be achieved by closed means. Postoperatively, all patients were immobilized in a long-arm splint with the forearm maintained in neutral rotation for 10–14 days for early soft-tissue protection.

### 2.3. Data Collection

Demographic and clinical data including age, sex, mechanism of injury, fracture localization along the radial shaft, time from injury to surgery, and operative duration were recorded. Surgical parameters included nail diameter, canal diameter, and the nail-to-canal diameter ratio. For subgroup analyses, fracture location was classified according to the level of the radial fracture on anteroposterior and lateral radiographs. The diaphyseal portion of the radius, bounded by the proximal and distal metaphyseal transition zones, was divided longitudinally into three equal segments. Fractures were categorized as proximal-third diaphyseal, middle-third diaphyseal, or distal-third diaphyseal according to the location of the midpoint of the radial fracture line. Thus, all included fractures were diaphyseal, and these categories represented subregions of the radial shaft rather than metaphyseal or physeal fracture locations.

### 2.4. Radiological Assessment

Early postoperative anteroposterior forearm radiographs were obtained during the standardized immobilization period, with the forearm maintained in neutral rotation. Because the children were in the early postoperative phase, with varying degrees of pain and soft-tissue swelling, removal of the splint and repositioning of the forearm in full supination solely for additional radiographic assessment was not part of the routine clinical protocol. Radial bowing was therefore evaluated on the consistently available neutral-rotation radiographs using an adaptation of the measurement principles described by Schemitsch and Richards [4]. The use of neutral-position radiographs for radial bow assessment has precedent in the literature. Bot et al. measured the magnitude and location of radial bow on standardized anteroposterior radiographs obtained with the forearm in the neutral position using the Schemitsch and Richards method [6]. Jayakumar and Jupiter similarly described radial bow assessment on anteroposterior forearm radiographs obtained in neutral rotation using a technique adapted from Schemitsch and Richards [7].

Because some published normative values were derived from radiographs obtained in supination, the MRB and LMRB thresholds used in the present study were considered predefined, literature-derived reference ranges rather than validated normative values for neutral-rotation radiographs. Simultaneous concordance with the predefined MRB and LMRB ranges was therefore evaluated as an exploratory composite radiographic outcome.

**Figure 1:**
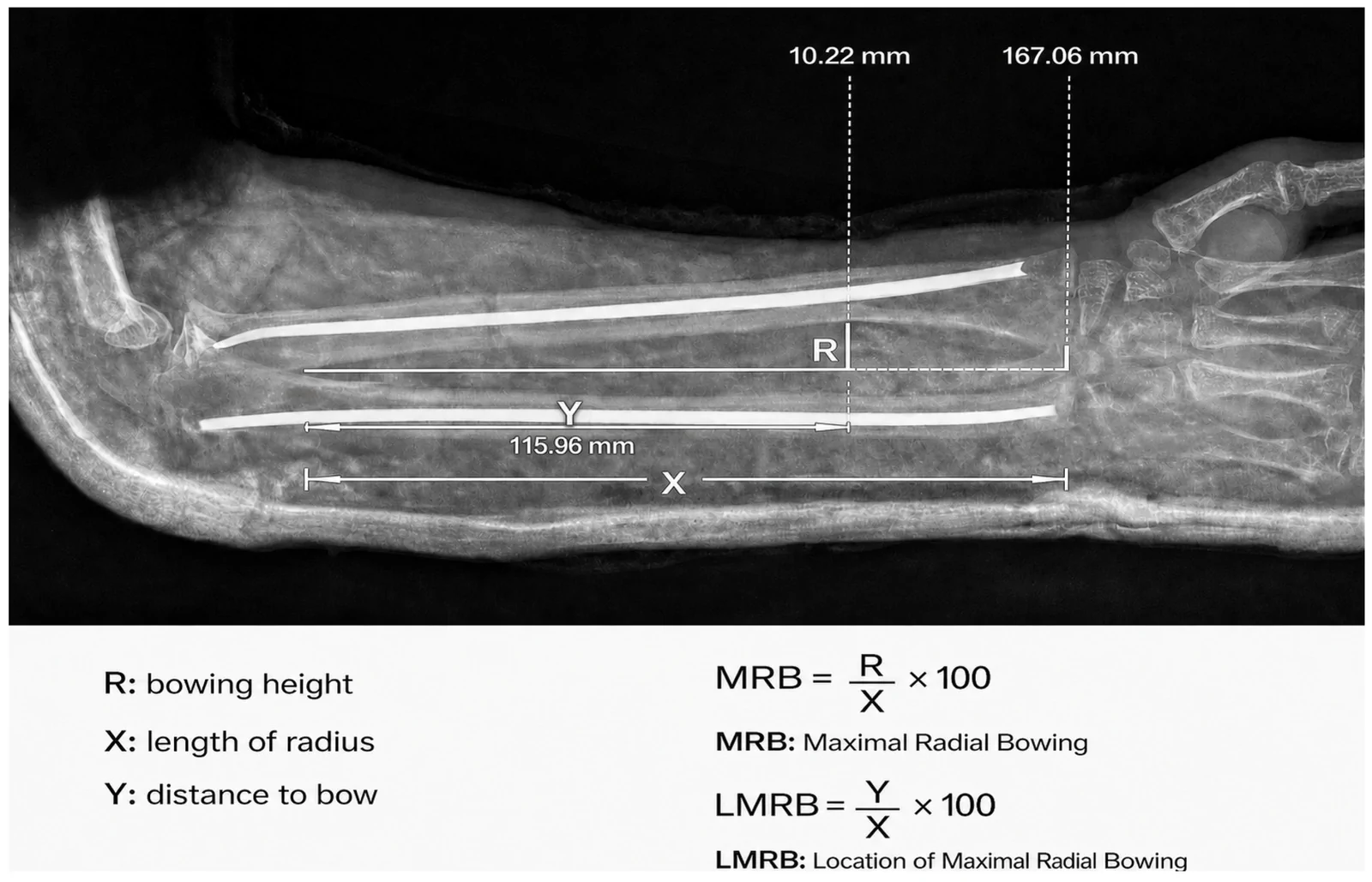
Schematic illustration of the radial bowing measurement technique applied on true anteroposterior forearm radiographs obtained in neutral rotation.

The amount of maximal radial bowing (MRB) was calculated as the ratio of the maximum perpendicular distance from the radial shaft to the longitudinal axis of the radius (R) divided by the total radial length (X), expressed as a percentage (MRB = R/X × 100). The location of maximal radial bowing (LMRB) was defined as the distance from the proximal reference point to the site of maximal bowing (Y) divided by the total radial length, also expressed as a percentage (LMRB = Y/X × 100) (Figure 1).

Based on previously reported reference values, MRB values of 6–9% and LMRB values of 55–75% of total radial length were predefined as the literature-derived reference ranges [3,5,8]. Because these thresholds were not specifically validated for neutral-rotation radiographs, simultaneous concordance with both ranges was evaluated as an exploratory composite radiographic outcome rather than as a definitive measure of anatomical restoration.

To further quantify deviation from the midpoint of the predefined MRB reference range, absolute MRB deviation was calculated as the absolute difference between the postoperative MRB value and 7.5%.

Additional radiographic parameters included coronal and sagittal angulation and postoperative translational displacement. Fracture union was defined as the presence of cortical continuity in at least three cortices on follow-up radiographs. All radiographic measurements, including MRB and LMRB, were performed jointly by two orthopaedic surgeons during the same assessment session. Both observers reviewed each radiograph, and a single final value was recorded for each parameter after consensus was reached. The measurements were not performed independently or repeated at separate time points.

### 2.5. Functional Assessment

Functional outcomes were assessed at final follow-up using forearm range-of-motion measurements, including pronation and supination. Overall clinical results were graded according to the Flynn criteria and categorized as excellent, good, fair, or poor. For statistical analysis, excellent and good outcomes were grouped together.

### 2.6. Statistical Analysis

All statistical analyses were performed using Jamovi statistical software (version 2.7.15; The Jamovi Project, Sydney, Australia). Continuous variables were assessed for normality using the Shapiro–Wilk test. Normally distributed variables were presented as mean ± standard deviation, whereas non-normally distributed variables were expressed as median with interquartile range (IQR). Categorical variables were presented as frequencies and percentages. Exploratory comparisons of demographic, surgical, and functional variables between patients who met and did not meet the composite radiographic definition were performed using the Mann–Whitney U test for continuous variables and the chi-square or Fisher’s exact test for categorical variables, as appropriate. MRB, LMRB, and MRB deviation were excluded from these inferential between-group comparisons because MRB and LMRB directly defined the grouping variable and MRB deviation was mathematically derived from MRB. Correlation analyses between radiographic parameters (MRB, LMRB, and MRB deviation) and functional outcomes (supination loss, pronation loss, total rotational loss, and Flynn category) were conducted using Spearman’s rank correlation coefficient. To evaluate the association between fracture characteristics and the exploratory composite radiographic outcome, subgroup analyses were performed according to radial diaphyseal fracture level (proximal third, middle third, and distal third). Multivariable logistic regression analysis was performed to identify factors independently associated with the exploratory composite radiographic outcome. Variables entered into the model included age, time from injury to surgery, nail-to-canal diameter ratio, surgical approach (open vs. closed), and radius fracture location. Surgical approach was recoded such that entries recorded as “no” were included in the closed-surgery group. A two-tailed *p*-value < 0.05 was considered statistically significant.

## 3. Results

A total of 61 pediatric patients treated with elastic stable intramedullary nailing for diaphyseal forearm fractures were included in the final analysis. The baseline demographic, radiological, and functional characteristics of the study population are summarized in Table 1. The mean age was 10.8 ± 2.6 years, and 53 patients (86.9%) were male. The mean maximal radial bowing (MRB) was 5.9% ± 0.9%. MRB was within the predefined literature-derived reference range of 6–9% in 34 patients (55.7%), whereas LMRB was within the predefined reference range of 55–75% in 52 patients (85.2%). Simultaneous concordance with both reference ranges was observed in 31 patients (50.8%) and was analyzed as an exploratory composite radiographic outcome. According to the Flynn criteria, 58 patients (95.1%) had excellent or good functional outcomes. The mean follow-up duration was 33.4 ± 13.2 months, with a median of 30.9 months and a range of 12.4–57.0 months.

**Figure 2:**
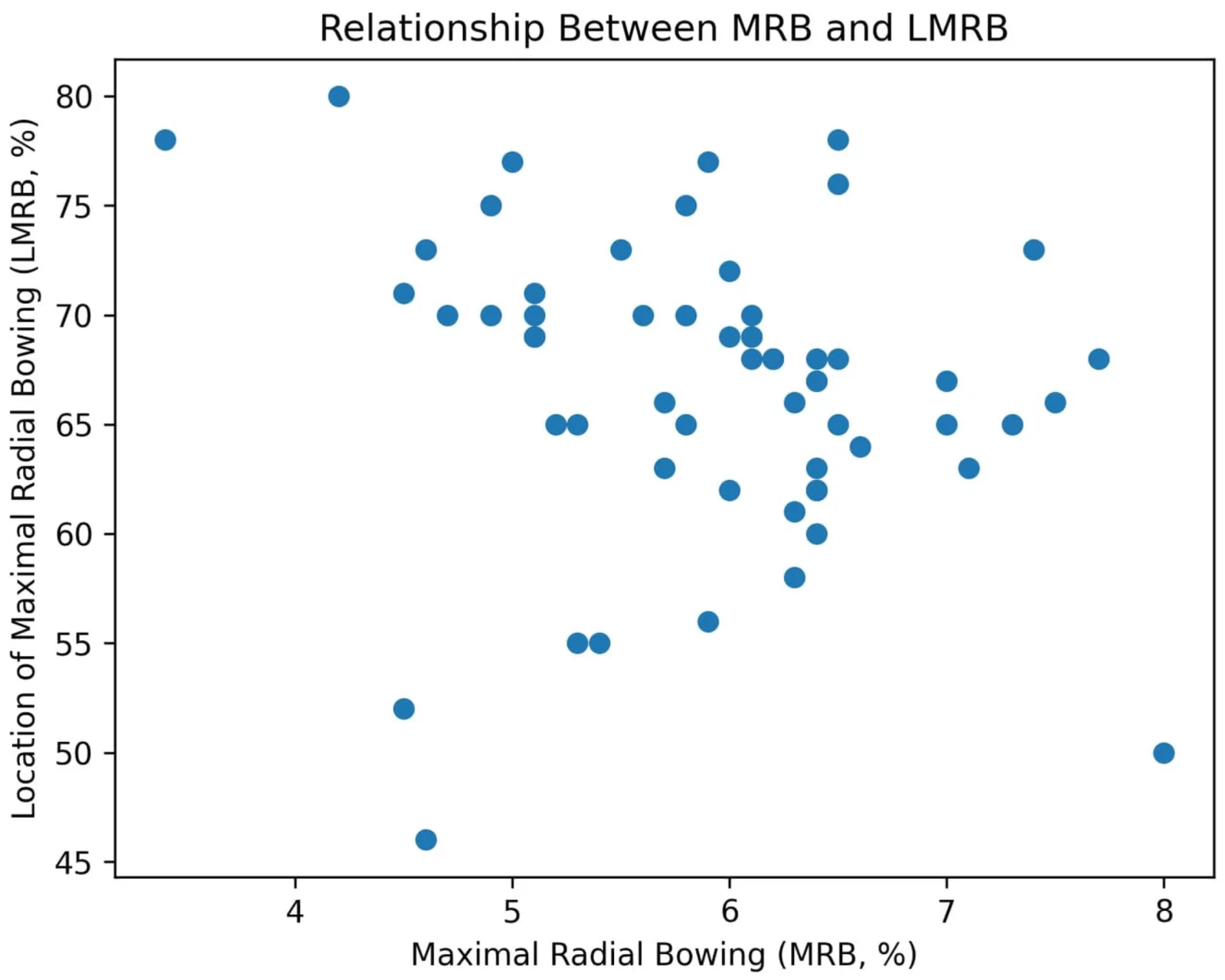
Scatter plot demonstrating the relationship between maximal radial bowing (MRB, %) and the location of maximal radial bowing (LMRB, %), showing a significant negative correlation.

Correlation analysis demonstrated a significant negative association between MRB and LMRB (Spearman rho = −0.30, *p* = 0.018), indicating that greater bowing magnitude was associated with a more proximally located maximal bow. This relationship is illustrated in Figure 2.

Exploratory comparisons of clinical, surgical, and functional variables according to the composite radiographic definition are presented in Table 2. Patients meeting the composite definition demonstrated less supination loss at final follow-up than those who did not meet the definition (*p* = 0.007). No statistically significant between-group differences were observed in pronation loss (*p* = 0.231) or total rotational loss (*p* = 0.061).

The association between fracture location and the exploratory composite radiographic outcome is presented in Table 3. Radial diaphyseal fracture level was significantly associated with the exploratory composite radiographic outcome (*p* = 0.018). Concordance rates were highest for proximal-third diaphyseal fractures (68.4%), followed by middle-third diaphyseal fractures (53.3%), and lowest for distal-third diaphyseal fractures (16.7%).

Spearman correlation analyses between the individual radiographic parameters and functional outcomes are summarized in Table 4. Neither MRB, LMRB, nor MRB deviation showed a statistically significant correlation with supination loss, pronation loss, total rotational loss, or Flynn category (all *p* > 0.05). Thus, no individual radiographic parameter demonstrated a significant monotonic association with any of the evaluated functional outcomes.

**Figure 3:**
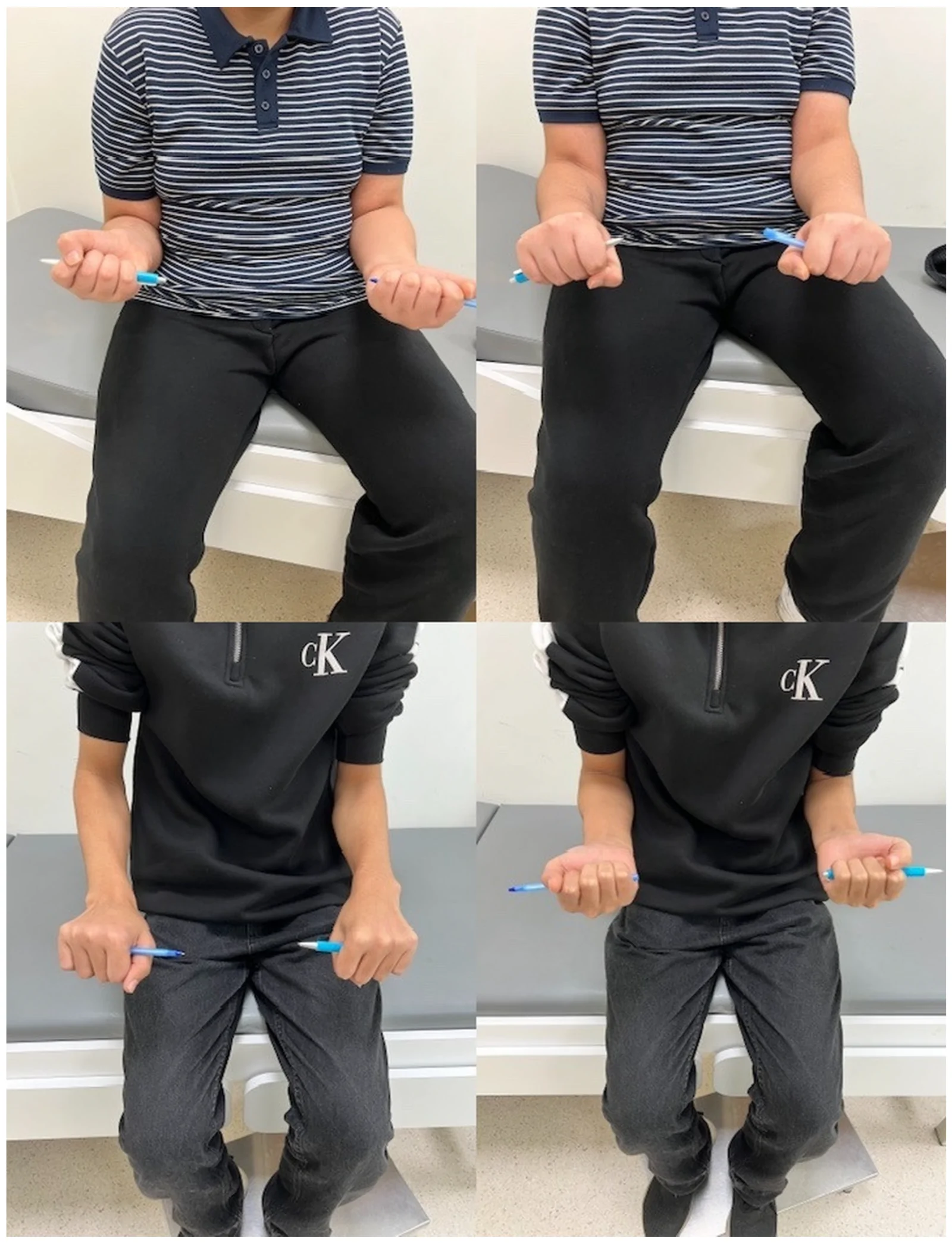
Clinical photographs demonstrating bilateral forearm rotational movements at final follow-up. The **upper row** shows one patient and the **lower row** shows another patient. In each row, supination is demonstrated on the left and pronation on the right, assessed using a handheld pen as a visual reference to illustrate symmetry and range of motion. These images highlight preserved forearm rotation following elastic stable intramedullary nailing.

Multivariable logistic regression analysis of factors associated with the exploratory composite radiographic outcome is presented in Table 5. Distal-third radial diaphyseal fracture location was associated with lower odds of meeting the composite radiographic definition (OR 0.18, 95% CI 0.03–1.09); however, this association did not reach statistical significance (*p* = 0.062). The injury-to-surgery interval was not independently associated with the composite radiographic outcome (OR 0.95 per day, 95% CI 0.87–1.03, *p* = 0.199). No independent associations were observed for age, nail-to-canal diameter ratio, or surgical approach. Representative clinical photographs demonstrating preserved forearm rotation are shown in Figure 3.

## 4. Discussion

The present study evaluated postoperative radial alignment in pediatric diaphyseal forearm fractures treated with elastic stable intramedullary nailing by jointly assessing the magnitude and anatomical location of maximal radial bowing. A combined evaluation of MRB and LMRB provided a broader radiographic description of postoperative radial alignment than bowing magnitude alone. Patients meeting the exploratory composite radiographic definition demonstrated less supination loss than those outside the predefined reference ranges. However, none of the individual radiographic parameters, including MRB, LMRB, and MRB deviation, showed a significant correlation with functional outcomes. The observed group difference therefore represents an association within a retrospective cohort and should not be interpreted as evidence that anatomical restoration directly resulted in improved forearm rotation.

The relationship between radiological alignment and clinical function in pediatric forearm fractures remains complex. Previous studies have reported inconsistent correlations between radial bowing parameters and forearm rotation, often demonstrating satisfactory functional outcomes despite suboptimal radiographic restoration [1,2,9]. In our study, although no significant monotonic correlation was found between individual radiographic parameters and pronation or supination, patients who met the exploratory composite radiographic definition demonstrated significantly less supination loss. The absence of significant correlations between the individual radiographic parameters and functional outcomes indicates that the relationship between radial alignment and forearm function is complex. Although the composite group difference in supination loss may warrant further investigation, the present findings do not establish that the combined construct is a superior functional predictor.

From a biomechanical perspective, radial bowing plays a critical role in optimizing the lever arm of the radius and maintaining efficient pronation–supination mechanics. Experimental studies have shown that alterations in radial curvature can significantly impair forearm rotation, particularly supination [10]. In this context, the lower supination loss observed among patients meeting the composite radiographic definition is compatible with the established biomechanical importance of radial curvature. However, the retrospective observational design does not allow us to determine whether the radiographic difference caused the observed functional difference.

An additional finding of this study was the association between fracture location and the exploratory composite radiographic outcome. Concordance rates were highest in proximal-third diaphyseal fractures and lowest in distal-third diaphyseal fractures. This observation may be explained by the inherent mechanical limitations of intramedullary fixation in distal segments, where achieving and maintaining optimal three-point fixation is more challenging. Previous clinical studies have suggested that distal forearm fractures may be associated with less predictable alignment following ESIN [11]. This finding suggests that fracture location may be relevant to postoperative radial alignment; however, the association requires confirmation in larger prospective cohorts. The observed difference between fracture locations appears to be primarily driven by the markedly lower concordance rates in distal-third diaphyseal fractures, whereas proximal and middle-third diaphyseal fractures showed relatively comparable outcomes.

The injury-to-surgery interval was not independently associated with the composite radiographic outcome in the multivariable analysis. Although the point estimate was compatible with higher odds of meeting the composite definition with a shorter interval, the confidence interval included the null value and the association was not statistically significant. This finding should therefore be interpreted cautiously and should not be used to infer a benefit of earlier intervention from the present data.

No independent association was observed between the nail-to-canal diameter ratio and the exploratory composite radiographic outcome. Although appropriate nail sizing is a fundamental principle of ESIN, modern surgical practice with standardized techniques may reduce variability in implant-related factors. Previous studies have emphasized the importance of proper nail contouring and balanced fixation rather than absolute nail size alone [11,12].

Importantly, none of the individual radiographic parameters—MRB, LMRB, or MRB deviation—showed a statistically significant correlation with supination loss, pronation loss, total rotational loss, or Flynn category. The lower supination loss observed among patients meeting the exploratory composite radiographic definition was derived from a dichotomized group comparison and should not be interpreted as evidence that any individual bowing parameter predicts function or that the composite definition is a validated or superior functional predictor. The absence of significant correlations may partly reflect the limited sample size and the restricted variability in functional outcomes, as 95.1% of the cohort had excellent or good results. Accordingly, the composite group difference should be regarded as hypothesis-generating and requires confirmation in larger prospective cohorts.

A recent study by Gundogdu et al. highlighted the importance of restoring radial bowing in pediatric forearm fractures and demonstrated its association with forearm rotation. While their findings provide important insight into the functional relevance of radial curvature, their analysis primarily focused on the magnitude of radial bowing [13]. In contrast, the present study expands on these findings by incorporating not only the magnitude but also the anatomical location of maximal radial bowing into a composite assessment. Our results suggest that evaluating these parameters in combination may provide a more comprehensive representation of forearm alignment than considering bowing magnitude alone. Furthermore, unlike previous studies, we analyzed the relationship between the exploratory composite radiographic outcome and both functional outcomes and surgical variables using multivariable modeling.

None of the individual radiographic parameters showed a statistically significant correlation with functional outcomes. Although patients meeting the exploratory composite radiographic definition had less supination loss, this group comparison does not establish that the combined construct is a superior functional predictor or that use of this assessment improves clinical outcomes.

Several limitations of this study should be acknowledged. First, the retrospective, single-center observational design introduces potential selection bias, measurement bias, and residual confounding and precludes causal inference. Consequently, the observed associations should not be interpreted as evidence that anatomical restoration directly improves forearm function or that consideration of MRB and LMRB during surgery improves treatment outcomes. Second, although objective functional measures such as range of motion were included, patient-reported outcome measures (PROMs) were not available, which may limit the assessment of subjective functional recovery. Third, the relatively small sample size may have limited the statistical power of multivariable analyses, increasing the risk of type II error. Fourth, radial bow measurements were obtained from neutral-rotation radiographs acquired during postoperative immobilization, whereas some published reference ranges were derived from supinated radiographs. Although neutral-position radial bow measurements have precedent in the literature and the consistent use of the same imaging protocol supports internal comparisons within our cohort, differences in forearm position may influence absolute MRB and LMRB values. Therefore, the literature-derived thresholds used in this study cannot be considered fully validated normative values for neutral-rotation radiographs. The composite outcome should consequently be interpreted as an exploratory measure of concordance with published reference ranges rather than definitive evidence of anatomical restoration. Fifth, the radiographic measurements were performed by consensus between two orthopaedic surgeons rather than through independent and repeated assessments. Consequently, formal interobserver and intraobserver reliability could not be calculated, and the reproducibility of the measurements remains to be established in future studies.

Despite these limitations, the present study provides a descriptive evaluation of postoperative radial bow magnitude and location following ESIN in pediatric forearm fractures. The observed difference in supination loss between the composite groups should be interpreted as an association and does not demonstrate that achieving or intraoperatively targeting the predefined MRB and LMRB ranges improves function or treatment outcomes. Prospective multicenter studies incorporating standardized imaging protocols, measurement reliability analyses, and patient-reported outcome measures are required to determine the clinical utility of the proposed exploratory composite radiographic outcome.

## 5. Conclusions

In this retrospective cohort of pediatric diaphyseal forearm fractures treated with ESIN, MRB and LMRB provided complementary descriptive information regarding postoperative radial alignment. Simultaneous concordance with the predefined literature-derived MRB and LMRB ranges was associated with less supination loss, whereas no individual radiographic parameter showed a significant correlation with functional outcomes. These findings support further evaluation of the exploratory composite radiographic outcome but do not establish that its use during surgery improves anatomical restoration, function, or fracture management.

## Figures and Tables

**Table 1:** Baseline demographic, radiological, and functional characteristics of the study population.

Parameter	Value
Number of patients	61
Age (years), mean ± SD	10.8 ± 2.6
Male sex, n (%)	53 (86.9)
Maximal radial bowing (MRB), mean ± SD	5.9 ± 0.9
MRB within predefined literature-derived reference range (6–9%), n (%)	34 (55.7)
LMRB within predefined literature-derived reference range (55–75%), n (%)	52 (85.2)
Simultaneous concordance with both reference ranges, n (%)	31 (50.8)
Flynn excellent or good, n (%)	58 (95.1)
Follow-up duration (months), mean ± SD	33.4 ± 13.2

Baseline demographic, radiological, and functional characteristics of the study population. MRB indicates maximal radial bowing; LMRB, location of maximal radial bowing.

**Table 2:** Comparison of clinical, surgical, and functional variables according to the exploratory composite radiographic outcome.

Variable	Met the Composite Definition (n = 31)	Did Not Meet the Composite Definition (n = 30)	*p* Value
Age (years), median (IQR)	11.0 (8.5–12.5)	12.0 (9.3–12.8)	0.788
Nail-to-canal diameter ratio, median (IQR)	0.48 (0.43–0.59)	0.44 (0.38–0.55)	0.390
Time from injury to surgery (days), median (IQR)	3.0 (1.0–5.5)	3.5 (2.0–13.8)	0.083
Operative time (minutes), median (IQR)	80.0 (65.0–100.0)	82.5 (62.5–93.8)	0.919
Supination loss (degrees), median (IQR)	0 (0–0)	0 (0–5)	0.007
Pronation loss (degrees), median (IQR)	0 (0–0)	0 (0–3.8)	0.231
Total rotational loss (degrees), median (IQR)	0 (0–0)	0 (0–10)	0.061

Comparison between patients who met and did not meet the exploratory composite radiographic definition. The composite definition required simultaneous concordance with the predefined literature-derived MRB (6–9%) and LMRB (55–75%) reference ranges. Continuous variables are presented as medians with interquartile ranges and were compared using the Mann–Whitney U test.

**Table 3:** Association between radial diaphyseal fracture level and the exploratory composite radiographic outcome.

Radial Diaphyseal Fracture Level	Met the Composite Definition (n = 31)	Did Not Meet the Composite Definition (n = 30)	Concordance Rate (%)	*p* Value
Proximal third of the radial diaphysis	13	6	68.4	
Middle third of the radial diaphysis	16	14	53.3	
Distal third of the radial diaphysis	2	10	16.7	0.018

Concordance with the exploratory composite radiographic definition according to radial diaphyseal fracture level. Distal-third diaphyseal fractures showed the lowest concordance rate. The p value was derived from the chi-square test; the overall difference was primarily driven by the low concordance rate in distal-third diaphyseal fractures.

**Table 4:** Correlation of radiographic parameters with functional outcomes.

Variable Pair	Spearman Rho	*p* Value
MRB vs supination loss	−0.183	0.157
MRB vs pronation loss	−0.103	0.431
MRB vs total rotational loss	−0.135	0.300
MRB vs Flynn category	−0.195	0.132
LMRB vs supination loss	0.115	0.377
LMRB vs pronation loss	0.005	0.967
LMRB vs total rotational loss	0.086	0.509
LMRB vs Flynn category	0.136	0.295
MRB deviation vs supination loss	0.203	0.117
MRB deviation vs pronation loss	0.118	0.365
MRB deviation vs total rotational loss	0.151	0.245
MRB deviation vs Flynn category	0.208	0.108

Correlations between radiographic bowing parameters and functional outcomes. No significant monotonic association was detected for any single radiographic parameter.

**Table 5:** Multivariable logistic regression for the exploratory composite radiographic outcome.

Variable	OR	95% CI	*p* Value
Closed surgery vs open surgery	0.98	0.30–3.20	0.978
Distal-third vs middle-third radial diaphyseal fracture	0.18	0.03–1.09	0.062
Proximal-third vs middle-third radial diaphyseal fracture	1.96	0.53–7.32	0.316
Age (per year)	1.01	0.80–1.28	0.938
Time from injury to surgery (per day)	0.95	0.87–1.03	0.199
Nail-to-canal diameter ratio	9.18	0.06–1514.55	0.395

## Data Availability

The data that support the findings of this study are available from the corresponding author upon reasonable request. The data are not publicly available because they contain information that could compromise the privacy of research participants.

## Abbreviations

ESIN	Elastic Stable Intramedullary Nailing
MRB	Maximal Radial Bowing
LMRB	Location of Maximal Radial Bowing
AP	Anteroposterior
IQR	Interquartile Range
CI	Confidence Interval
OR	Odds Ratio
PROMs	Patient-Reported Outcome Measures
